# Gait Metrics in Elderly Fallers and Non-Fallers with Varying Levels of Glaucoma: A Longitudinal Prospective Cohort Study

**DOI:** 10.3390/s25123712

**Published:** 2025-06-13

**Authors:** Louay Almidani, José G. Vargas, Zhuochen Yuan, Seema Banerjee, Xindi Chen, Mariah Diaz, Rhonda Miller, Aleksandra Mihailovic, Pradeep Y. Ramulu

**Affiliations:** 1 Wilmer Eye Institute, Johns Hopkins University School of Medicine, Baltimore, MD 21287, USA; 2 College of Medicine, University of Illinois Chicago, Chicago, IL 60612, USA

**Keywords:** gait changes, longitudinal, falls, older adults

## Abstract

To understand the impact of falls on gait in those with poor sight, we examined how gait changed after falls in older adults with varying degrees of visual impairment from glaucoma. Participants were classified as fallers or non-fallers based on prospective falls data from the first study year. Injurious fallers were those who suffered injuries from falls. The GAITRite Electronic Walkway characterized gait at baseline and three annual follow-ups. Parameters examined included stride length, variability in stride length (CV), stride velocity, stride velocity CV, base of support, base of support CV, and cadence. Longitudinal gait changes were assessed using generalized estimating equation models. Stride length significantly decreased in both fallers (β = −0.09 z-score unit/year) and non-fallers (β = −0.08 z-score unit/year), stride velocity slowed only among fallers (β = −0.08 z-score unit/year), and, in contrast, stride velocity CV decreased only among non-fallers (β = −0.07 z-score unit/year). No longitudinal differences were noted between groups. Additionally, no significant differences in gait metrics were observed between non-fallers, one-time fallers, and multiple fallers, nor between those with and without an injurious fall. Amongst older adults, and enriched for those with visual impairment, fallers and non-fallers adopted a more cautious gait over time, with similar gait changes across groups. Our results suggest that, in visual impairment, many falls may not lead to significant changes in gait.

## 1. Introduction

Gait disorders are frequent in older adults, and their prevalence increases with age, reaching over 60% in patients aged 80 years or above [1]. Gait disorders and several gait parameters are linked with falls [2,3,4], however, as we have previously shown, this relationship is more complex, with the relationship depending on whether falls were evaluated as a rate over time or over activity (falls/year or falls/step) [5,6].

Given the significant consequences of falls and the importance of preventing falls, identifying modifiable risk factors is necessary [7,8]. Numerous non-gait-based metrics, including fear of falling, balance assessment, and physical activity, have been studied as potential modifiable risk factors for falls [9,10,11]. For instance, we have previously shown that multiple falls are associated with longitudinal declines in physical activity measured using a waist-bound accelerometer [12]. Since several clinical practice guidelines suggest gait assessment to better understand fall risk, we aimed to build upon our previous work and explore whether gait metrics vary by fall [13]. This is particularly important because gait changes are an even greater risk factor for falls compared to physical activity, with a recent review by Colón-Emeric and colleagues (2024) suggesting over a two-fold increased risk of falls in those with gait abnormalities [9].

Further, previous studies have suggested that gait changes associated with falls are reflective of more cautious walking [14]. However, these studies evaluated falls retrospectively, were cross-sectional in design, or had limited follow-up time or gait metrics assessed [14,15,16,17,18,19,20,21]. Since retrospective fall assessment correlates poorly with prospective fall documentation, longitudinal data is necessary to identify gait changes associated with falls [22]. To our knowledge, no studies have assessed whether prospectively recorded falls influence changes in gait patterns in patients with visual impairment over an extended duration.

In this study, we analyzed data from a three-year prospective observational study, the Falls In Glaucoma Study, to investigate the longitudinal changes in gait between individuals who experienced falls (fallers) and those who did not (non-fallers) in adults with varying levels of glaucomatous damage, and whether falls lead to gait changes reflective of more cautious walking. By prospectively identifying falls (including their number and severity) and tracking gait trajectories, our study is uniquely designed to elucidate whether falls influence changes in gait patterns. We hypothesized that fallers, particularly those who sustained injuries (injurious fallers), would exhibit changes in gait metrics during subsequent follow-up visits compared to their counterparts who did not experience any falls.

## 2. Methods

### 2.1. Study Design and Study Population

This observational cohort study included participants from a prospective longitudinal study, the Falls in Glaucoma Study (FIGS), conducted at the Johns Hopkins Wilmer Eye Institute on subjects enrolled from 2013 to 2015. Details have been described previously [6,23,24]. Briefly, eligibility criteria included the following: (1) age 60 or older during the study period; (2) diagnosis of suspect glaucoma or glaucoma not secondary to another systemic condition; (3) residence within a 60-mile radius from the Wilmer Eye Institute; and (4) ability to perform visual field (VF) testing. Exclusion criteria were the following: (1) presence of visually significant concurrent eye disease; (2) any ocular or non-ocular surgery in the past two months; (3) any hospitalization in the past month; (4) confinement to a bed or wheelchair; and (5) history of stroke or other neurological disorders causing VF damage. Supplemental Figure S1 describes the timeline of the study.

This study was conducted in accordance with the tenets of the Declaration of Helsinki and was approved by the Johns Hopkins University School of Medicine Institutional Review Board. All participants gave written consent. The study adheres to the Strengthening the Reporting of Observational Studies in Epidemiology (STROBE) reporting guidelines.

### 2.2. Gait Evaluation

Gait data were collected at baseline and annually during follow-up evaluations using the GAITRite Electronic Walkway (CIR Systems, Inc., Franklin, NJ, USA) [25,26,27,28]. The GAITRite system measures temporal and spatial gait parameters via an electronic walkway, and has been validated against a motion analysis system, demonstrating highly reliable measurements of temporospatial gait parameters [28,29,30].

Participants’ gait measurements were collected during a normal-paced barefoot walk. In each study year, participants wore their regular distance eyeglasses and were instructed to walk naturally for four full lengths of the mat (back and forth two times, with a short pause between each walk). This analysis focused on the following gait parameters, which prior research suggested may be relevant to falls: velocity, cadence, base of support, and stride length [31,32,33].

Additionally, stride-to-stride variability, which has been linked to fall rates, was assessed by calculating the coefficients of variation (ratio of the standard deviation to the mean multiplied by 100) for stride length, stride velocity, and base of support [34]. Further details have been described previously [6].

### 2.3. Falls Data Collection

Falls data were collected for each participant (*n* = 240) over the 36 months of the study, with the current study focusing on data collected within the first 12 months of the study. A fall was defined as unintentionally coming to rest on the ground or a lower level [6]. Study participants were provided with the definition and an instructional video for further illustration [35]. Subjects were also provided paper calendars to record falls, which were returned/reported monthly via mail or email. If fall calendars were not received, participants were contacted by phone and/or email to collect up to three months of missing data. Falls data not collected within a three-months period were recorded as missing.

### 2.4. Visual Assessment

VF tests were evaluated using the Humphrey Field Analyzer II (Carl Zeiss Meditec, Inc., Dublin, CA, USA) as described by Odden et al. [36]. Tests were obtained at the baseline study visit or at a recent clinic visit (median time = 2.5 months) for each eye separately and combined to create integrated VF (IVF) sensitivity. IVF sensitivity was calculated by combining pointwise sensitivities from both eyes to generate a sensitivity at each spatial coordinate using the maximum sensitivity approach [23]. Distance visual acuity was assessed using a back-lit Early Treatment Diabetic Retinopathy Study chart placed at a 4 m distance. If applicable, patients were instructed to wear their distance eyeglasses. Contrast sensitivity was evaluated using the Mars Letter Contrast Sensitivity Test (Mars Perceptrix Corporation, New Castle, NY, USA) [37], with participants wearing their usual eyeglasses. Distance visual acuity was calculated as the logarithm of the minimum angle of resolution (logMAR), while contrast sensitivity was measured in logCS units.

### 2.5. Evaluation of Covariates

Participants’ demographic characteristics were collected using standardized questionnaires. To assess for comorbid illnesses, a questionnaire was deployed that asked subjects if they had been diagnosed with any of 15 distinct comorbid illnesses: arthritis, broken or fractured hip, back problems, history of heart attack, history of angina/chest pain, congestive heart failure, peripheral vascular disease, high blood pressure, diabetes, emphysema, asthma, stroke, Parkinson’s disease, cancer other than skin cancer, and history of vertigo or Meniere’s disease. Total comorbidities were summed. Participants with more than five comorbid illnesses (*n* = 9) were reclassified as having five comorbidities. Medication data were collected by directly observing medication bottles, or otherwise by patient report. Patients were classified as having polypharmacy if they took five or more daily prescription medications, excluding eye drops.

### 2.6. Statistical Analysis

Descriptive statistics were used to compare demographics and health characteristics of the study population by faller status. Comparisons were performed using Pearson’s chi-squared test for categorical variables and t-test for continuous variables.

The main exposure variable was fall status (non-faller vs. faller; non-faller vs. single faller vs. multiple faller; or injurious faller vs. absence of an injurious fall). For gait, there were two different sets of outcome variables: parameter values (metrics such as velocity, cadence, or base of support) and coefficients of variation (CV), expressed as a percentage and calculated by dividing the standard deviation by the mean value of a parameter, to evaluate the extent of gait variability. For comparison purposes, all gait metrics were converted to z-score units as follows: (individual patient value − average study sample value)/(study sample standard deviation), with study sample values/variance taken from baseline visits.

In longitudinal analyses, we explored how gait metrics changed over time. Change rates were derived from models that utilized generalized estimating equations to explore how gait metrics varied over a three-year period, accounting for multiple observations per person. An interaction term between falls and visit was included to explore how change rates differed by faller status. Further, to explore how baseline factors predict changes in gait metrics, change rates over time in each gait metric were computed separately using individual linear regression models. Subsequent regression models were employed to explore predictors of change rates in gait metrics independent of faller status. All models were adjusted for age, race, sex, IVF, comorbidities, and polypharmacy. “Don’t know” and “Refuse” responses were treated as missing values and excluded from the regressions. Statistical significance was defined at *p* < 0.05. All *p* values were two-sided but not adjusted for multiple analyses. All analyses were conducted using STATA 16.0 (StataCorp LP, College Station, TX, USA).

## 3. Results

A total of 240 participants were included in the study, with a mean age of 71. Approximately half of the participants were female, one-third were African American, 44% were classified as fallers, 17% had multiple falls, and 21% had an injurious fall. Over the first 12 months of follow-up for each participant, the cumulative probability of experiencing one or more falls was 44.8%, and the cumulative probability of experiencing two or more falls was 17.7%.

Baseline characteristics were similar between the fallers and non-fallers groups (Table 1). In pairwise comparisons, stride length significantly (*p* = 0.03) differed between one-time fallers (mean [SD] = 108.32 [15.6]) vs. non-fallers (mean [SD] = 113.67 [16.5]), and between multiple fallers (mean [SD] = 116.94 [16.0]) and one-time fallers (*p* = 0.009). The base of support CV significantly differed between multiple fallers (mean [SD] = 27.09 [13.4]) and one-time fallers (mean [SD] = 21.73 [12.4]); *p* = 0.04. The remaining baseline variables did not significantly differ between the three groups (*p* > 0.05). When comparing injurious fallers to non-injurious fallers, injurious fallers (80.4%) were more likely to have >1 comorbidity compared to non-injurious fallers (61.4%); *p* = 0.01. The remaining baseline characteristics did not significantly differ between the groups (*p* > 0.05).

In both fallers and non-fallers, stride length decreased over time (fallers: β = −0.08 z-score unit/year, non-fallers: β = −0.09 z-score unit/year, *p* < 0.001 for both). Fallers displayed a decline in stride velocity over time (β = −0.08 z-score unit/year, *p* = 0.003), while non-fallers exhibited reduced variability in stride velocity (β = −0.07 z-score unit/year, *p* = 0.009). However, there were no significant changes in the remaining gait metrics (base of support, cadence, base of support CV, and stride length CV) for both groups throughout the study period (*p* > 0.05). When comparing fallers and non-fallers, the rates of change in gait metrics did not significantly differ between groups (*p* > 0.05). Supplemental Figures S2–S4 show the average stride length, velocity, and cadence in standardized z-scores by faller status over the three-year follow-up period.

Table 2 shows the associations between gait changes (in standardized z-scores) with non-fallers, one-time fallers, and multiple fallers. Stride length decreased in all three groups compared to baseline (non-fallers: β = −0.08, [95%CI: −0.11, −0.04], one-time fallers: β = −0.07, [95%CI: −0.12, −0.02], multiple fallers: β = −0.12, [95%CI: −0.18, −0.06]), while the remaining gait metrics did not significantly change over time (*p* > 0.05). No significant differences in gait metrics between the groups were found in pairwise comparisons to the non-faller group (*p* > 0.05).

Similarly, when examining longitudinal gait changes in those with or without an injurious fall, both injurious fallers (β = −0.08, [95% CI: −0.14, −0.03]) and those without an injurious fall (β = −0.08, [95% CI: −0.11, −0.05]) demonstrated decreased stride length over time. Injurious fallers, but not those without an injurious fall, also exhibited an increased base of support (β = 0.06, [95%CI: 0.003, 0.11]), while non-injurious fallers exhibited decreased velocity (β = −0.06, [95% CI: −0.10, −0.02]) and velocity CV (β = −0.07, [95% CI: −0.12, −0.02]). The remaining gait metrics did not significantly change over time in either group (*p* > 0.05). When comparing changes in gait metrics between injurious fallers and those without an injurious fall, no significant differences in gait metrics were observed between the groups (Table 3).

When exploring factors that predicted more rapid change in gait metrics independent of faller status (Table 4), worse baseline IVF was significantly associated with declines in stride velocity (β = −0.05 z-score unit/year, [95% CI: −0.11, −0.004]; per 5 dB increment) and cadence (β = −0.08 z-score unit/year, [95% CI: −0.13, −0.02]; per 5 dB increment) over time, while age was significantly associated with decreased cadence over time (β = −0.01 z-score unit/year, [95% CI: −0.02, −0.002]; per year older). The remaining associations were not significant (*p* > 0.05).

## 4. Discussion

In a sample of community-dwelling elderly adults at high risk of falls due to enrichment for visual impairment, we examined if falls and/or injurious falls were associated with specific longitudinal gait changes. Surprisingly, even across a full battery of gait metrics, changes in gait over time were not associated with fall status among fallers, multiple fallers (as compared to non-fallers), or even injurious fallers (compared to those with no injurious fall). These findings suggest that most falls may not result in longitudinal changes in gait in older adults.

Our finding that falls were not associated with changes in gait is somewhat at odds with previous studies which have shown associations between gait parameters and fall status [14,33]. However, these prior studies did not include repeated measurements of gait over a long follow-up period, though some did collect falls data prospectively. The discordance could thus be attributed to these fundamental differences in methodology, though other factors may contribute as well, including differences in the demographic characteristics of the study populations or the different tendencies of each population to adjust their activity levels in response to falling [6]. While previous studies have primarily focused on limited gait metrics, our study utilized an objective measurement of a comprehensive battery of gait metrics, offering more valuable insights. Prospective identification of falls is considered the gold standard for fall identification; indeed Howcroft et al. (2018) [21] demonstrated that gait differences between fallers and non-fallers were dependent on whether the classification of fallers was based on retrospective or prospective fall occurrence, which could explain the discrepancy between our results and those that relied on retrospective examination of falls [14].

There are several possibilities on why falls were not associated with changes in gait in our study. One possibility is that only very severe falls or those that cause a meaningful injury affect gait changes, and over the study period, few would have experienced such falls. Another possibility is that varying levels of fear of falling may develop as a result of a fall, leading to paradoxical differences in gait metrics. Further, long-standing gait habits may require longer monitoring periods to observe changes in different populations. The directionality of the relationship is important. It may be that changes in gait lead to falls rather than vice versa. Overall, gait changes related to various fall types (one-time falls, multiple falls, and injurious falls) are complex and require further research with longer observation periods across different populations.

We further explored predictors of gait changes over time and found that the severity of visual field damage, as well as age, predicted change rates in specific gait metrics, while a comorbidity index and polypharmacy did not. This finding agrees with our previous work and suggests that visual field damage results in declines in specific gait measures greater than that noted for several comorbidities [38].

Here, we focused on gait metrics due to their significant role as a risk factor for falls. However, it is also important to consider non-gait-based metrics. For example, we have previously shown that multiple fallers may be at high risk for a decline in physical capacity and endurance [12]. Other factors related to frailty, such as muscle weakness and self-reported exhaustion, may be potential targets for fall prevention, with previous trials demonstrating that improving leg strength and balance can help reduce the risk of falls [9,39].

Our study has a few limitations. First, gait was assessed barefoot and under controlled conditions (flat surface, without any obstructions, and good ambient lighting), which is not representative of the environment in which individuals usually walk. Previous work has noted gait degradation depending on lighting/environmental conditions and thus, our results might be underestimating the relationship between falls and gait changes over time, which may be more apparent as patients navigate increasingly complex environments [40]. Second, examination may have also been affected by the Hawthorne effect, in which non-fallers may be fall-aware and thus walk more cautiously [41]. Third, since we did not assess previous fall history, participants may have already undergone gait adaptation due to prior falls [42]. This idea is supported by research by Mansfield et al. (2015), which shows that fallers with previous history of fall shows notable modifications in gait mechanics [43]. Fourth, as a single center with strict inclusion criteria, the generalizability of the results may be limited. Fifth, fall history details were based on self-reports, which may introduce recall bias. Sixth, the study was originally powered to detect changes in fall rates by severity of visual field damage. It is possible that our sample in the presented analysis may be underpowered to detect gait changes by faller status. Further, it is important to consider physical activity when examining both falls and gait parameters [5]. Previous works have shown that several gait parameters were not significantly associated with falls per time but were significantly associated with falls per step [5]. For this specific analytic question, we did not incorporate physical activity into our models, as we were more interested to understand the effect of an actual fall on the gait changes. However, as gait metrics are most relevant to falls during active states such as walking, it is particularly important to assess gait metrics as a risk factor for falls in models that incorporate activity when falls are considered the outcome (as opposed to the exposure) [6]. With advancements in technology, long-term gait monitoring in daily life is warranted to better understand and mitigate fall risk [44,45].

## 5. Conclusions

In this three-year prospective observational study of community-dwelling elderly adults at high risk of falls due to visual impairment, a full battery of gait metrics did not show differences in gait changes over time across fall status, including multiple falls or injurious falls. This may be due to the complex and potentially bidirectional relationship between gait and falls, and the need for longer follow-up to detect subtle changes. Overall, these findings suggest that many falls may not lead to longitudinal changes in gait in older adults with visual impairment. Study limitations include controlled testing environment, unmeasured prior fall history, and limited generalizability. Future studies with longer observational time are needed to further evaluate this relationship.

## Figures and Tables

**Table 1:** Baseline participant characteristics, vision, and gait metrics by first year faller status (N = 240). Abbreviations; SD: standard deviation; Polypharmacy: ≥5 systemic prescription medications; IVF: integrated vision field; dB: decibels; IQR: interquartile range; MD: mean deviation; VA: visual acuity; logMAR: logarithm of the minimum angle of resolution; CS: contrast sensitivity; logCS: logarithm contrast sensitivity; CV: coefficient of variation.

	Non-Faller N = 135	Faller N = 105	*p*-Value
Demographics and health			
Age (year), mean (SD)	69.6 (7.73)	71.8 (7.36)	0.03
Female, *n* (%)	58 (43)	57 (54)	0.08
African American, *n* (%)	43 (32)	26 (25)	0.23
Living alone, *n* (%)	23 (17)	24 (23)	0.26
Polypharmacy, *n* (%)	41 (30)	38 (36)	0.34
No. of comorbidities			
>1, *n* (%)	81 (60)	76 (72)	0.05
Vision			
IVF sensitivity (dB), median (IQR)	28.1 (26.3, 29.9)	27.8 (25.7, 29.3)	0.19
MD better eye (dB), median (IQR)	−2.4 (−4.9, −0.5)	−3.0 (−5.6, −1.0)	0.19
MD worse eye (dB), median (IQR)	−6.0 (−13.6, −2.9)	−5.5 (−11.4, −2.8)	0.64
VA better eye (logMAR), median (IQR)	0.06 (0.0, 0.2)	0.06 (−0.02, 0.14)	0.52
CS both eyes (logCS), median (IQR)	1.72 (1.60, 1.76)	1.72 (1.64, 1.76)	0.60
Gait			
Base of support (cm), mean (SD)	10.02 (3.12)	10.41 (3.13)	0.34
Base of support CV (%), mean (SD)	24.98 (12.97)	23.77 (12.98)	0.47
Stride length (cm), mean (SD)	113.67 (16.51)	111.61 (16.25)	0.33
Stride length CV (%), mean (SD)	4.60 (2.35)	4.98 (3.21)	0.30
Stride velocity (cm/s), mean (SD)	101.33 (19.31)	100.47 (17.58)	0.72
Stride velocity CV (%), mean (SD)	6.90 (3.37)	7.29 (4.45)	0.44
Cadence (steps/min), mean (SD)	106.17 (10.43)	107.28 (10.66)	0.42

**Table 2:** The relationship between multiple fallers with changes in gait metrics over time.

		Base of Support		Stride Length		Stride Velocity		Cadence	
Predictor	Comparator/Reference	β	95% CI	β	95% CI	β	95% CI	β	95% CI
Fall status									
One-time faller	Non-faller	0.24	(−0.06, 0.53)	−0.10	(−0.38, 0.17)	0.00	(−0.30, 0.30)	0.17	(−0.15, 0.49)
Multiple faller	Non-faller	−0.05	(−0.40, 0.30)	0.36	(0.04, 0.69)	0.38	(0.03, 0.73)	0.21	(−0.17, 0.59)
Visit	Per visit	0.03	(0, 0.07)	−0.07 **	(−0.11, −0.04)	−0.05	(−0.09, 0)	0.01	(−0.03, 0.06)
Age	1 year older	0.01	(−0.01, 0.02)	−0.04 **	(−0.05, −0.03)	−0.04 **	(−0.05, −0.03)	−0.02	(−0.03, 0)
Male	Female	0.42 **	(0.20, 0.65)	0.71 **	(0.51, 0.92)	0.31 **	(0.11, 0.51)	−0.40 **	(−0.63, −0.18)
African American	White	−0.03	(−0.30, 0.23)	−0.31 *	(−0.55, −0.08)	−0.40 **	(−0.64, −0.16)	−0.27	(−0.53, 0)
IVF	5 dB decrement	0.22 **	(0.09, 0.35)	−0.16 *	(−0.28, −0.04)	−0.15 *	(−0.27, −0.03)	−0.06	(−0.2, 0.07)
Comorbidity (>1)	0–1	0.27 *	(0.02, 0.53)	−0.32 *	(−0.55, −0.09)	−0.33 *	(−0.56, −0.10)	−0.17	(−0.43, 0.08)
Polypharmacy	No polypharmacy	−0.02	(−0.29, 0.25)	−0.17	(−0.41, 0.06)	−0.16	(−0.40, 0.08)	−0.05	(−0.32, 0.21)
Fall status × Visit									
One-time faller	Non-faller	−0.02	(−0.08, 0.04)	0.00	(−0.06, 0.07)	−0.01	(−0.09, 0.07)	−0.03	(−0.11, 0.05)
Multiple faller	Non-faller	−0.001	(−0.07, 0.07)	−0.04	(−0.11, 0.03)	−0.06	(−0.15, 0.03)	−0.04	(−0.13, 0.06)

CI: confidence interval; dB: decibel; IVF: integrated visual field. * *p* < 0.05, ** *p* < 0.001.

**Table 3:** The relationship between injurious fallers with changes in gait metrics over time.

		Base of Support		Stride Length		Stride Velocity		Cadence	
Predictor	Comparator/Reference	β	95% CI	β	95% CI	β	95% CI	β	95% CI
Fall status									
Injurious faller	Non-injurious faller	0.03	(−0.28, 0.34)	0.26	(−0.03, 0.54)	0.36 *	(0.06, 0.67)	0.31	(−0.02, 0.64)
Visit	Per visit	0.02	(−0.01, 0.05)	−0.08 **	(−0.11, −0.05)	−0.06 *	(−0.10, −0.02)	−0.01	(−0.05, 0.03)
Age	1 year older	0.01	(−0.01, 0.02)	−0.04 **	(−0.05, −0.03)	−0.04 **	(−0.05, −0.03)	−0.02 *	(−0.03, −0.003)
Male	Female	0.41 **	(0.18, 0.63)	0.73 **	(0.53, 0.93)	0.32 **	(0.12, 0.52)	−0.40 **	(−0.62, −0.18)
African American	White	−0.02	(−0.28, 0.25)	−0.32 *	(−0.56, −0.08)	−0.39 **	(−0.62, −0.15)	−0.24	(−0.50, 0.02)
IVF	5 dB decrement	0.22 **	(0.09, 0.35)	−0.16 *	(−0.28, −0.04)	−0.15 *	(−0.27, −0.03)	−0.06	(−0.19, 0.07)
Comorbidity (>1)	0–1	0.27 *	(0.01, 0.53)	−0.34 *	(−0.58, −0.11)	−0.37 **	(−0.60, −0.14)	−0.22	(−0.47, 0.04)
Polypharmacy	No polypharmacy	−0.004	(−0.27, 0.26)	−0.20	(−0.43, 0.04)	−0.18	(−0.41, 0.06)	−0.05	(−0.31, 0.21)
Fall status × Visit									
Injurious faller	Non-injurious faller	0.04	(−0.03, 0.10)	−0.01	(−0.07, 0.06)	0.003	(−0.08, 0.09)	0.04	(−0.05, 0.12)

CI: confidence interval; dB: decibel; IVF: integrated visual field. * *p* < 0.05, ** *p* < 0.001.

**Table 4:** Predictors of change rates in gait metrics (in standardized z-scores/year).

		Base of Support		Stride Length		Stride Velocity		Cadence	
Predictor	Comparator/Reference	β	95% CI	β	95% CI	β	95% CI	β	95% CI
Fall status									
Faller	Non-faller	−0.01	(−0.08, 0.06)	−0.01	(−0.08, 0.06)	−0.03	(−0.12, 0.06)	−0.04	(−0.14, 0.05)
Age	1 year older	0.004	(0, 0.01)	−0.004	(−0.01, 0)	−0.01	(−0.01, 0)	−0.01 *	(−0.02, −0.002)
Male	Female	0.03	(−0.03, 0.10)	−0.003	(−0.07, 0.07)	0.02	(−0.06, 0.11)	0.05	(−0.04, 0.14)
African American	White	−0.06	(−0.14, 0.02)	0.02	(−0.06, 0.11)	0.02	(−0.08, 0.12)	0.003	(−0.11, 0.11)
IVF	5 dB decrement	0.02	(−0.02, 0.06)	−0.03	(−0.07, 0.01)	−0.05 *	(−0.11, −0.004)	−0.08 *	(−0.13, −0.02)
Comorbidity (>1)	0–1	0.06	(−0.01, 0.14)	−0.04	(−0.12, 0.04)	−0.05	(−0.15, 0.05)	−0.03	(−0.13, 0.07)
Polypharmacy	No polypharmacy	−0.06	(−0.14, 0.02)	0.07	(−0.02, 0.15)	0.08	(−0.02, 0.18)	0.07	(−0.04, 0.18)

CI: confidence interval; dB: decibel; IVF: integrated visual field. * *p* < 0.05.

## Data Availability

Data will be made available upon reasonable request to the corresponding author.

## Supplementary Materials

The following supporting information can be downloaded at: https://www.mdpi.com/article/10.3390/s25123712/s1, Figure S1. Timeline of the study. Figure S2. Average stride length in standardized z-scores by faller status over the three-year follow-up period. Figure S3. Average stride velocity in standardized z-scores by faller status over the three-year follow-up period. Figure S4. Average cadence in standardized z-scores by faller status over the three-year follow-up period.
