# Supplementary figures and images for: Gait Metrics in Elderly Fallers and Non-Fallers with Varying Levels of Glaucoma: A Longitudinal Prospective Cohort Study
Source: Sensors (Basel). 2025 Jun 13;25(12):3712. doi: 10.3390/s25123712 (PMC12197231; doi:10.3390/s25123712)

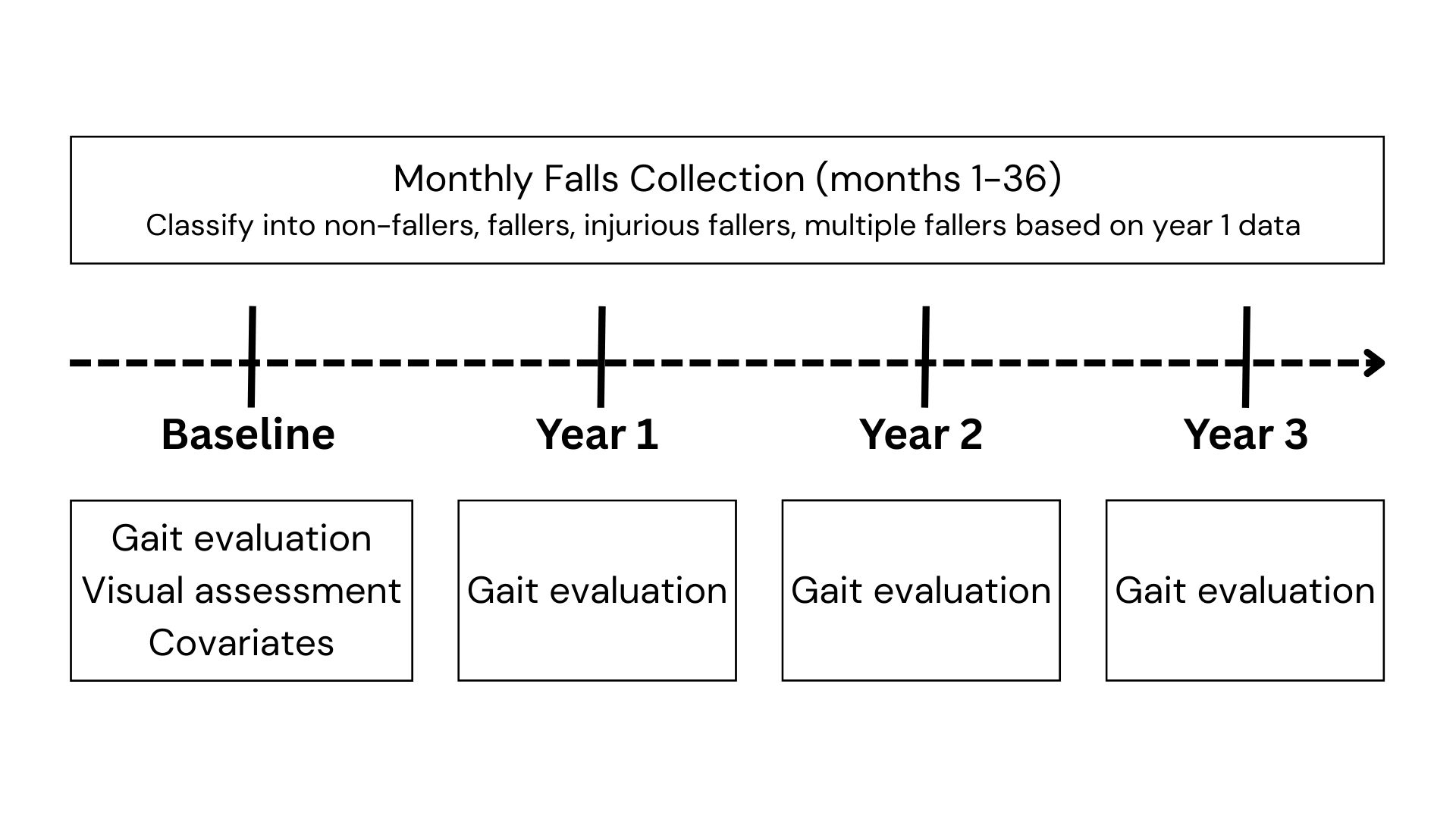

Supplement: Supplementary file 1 [file sensors-25-03712-s001.zip › FigureS1.jpg]

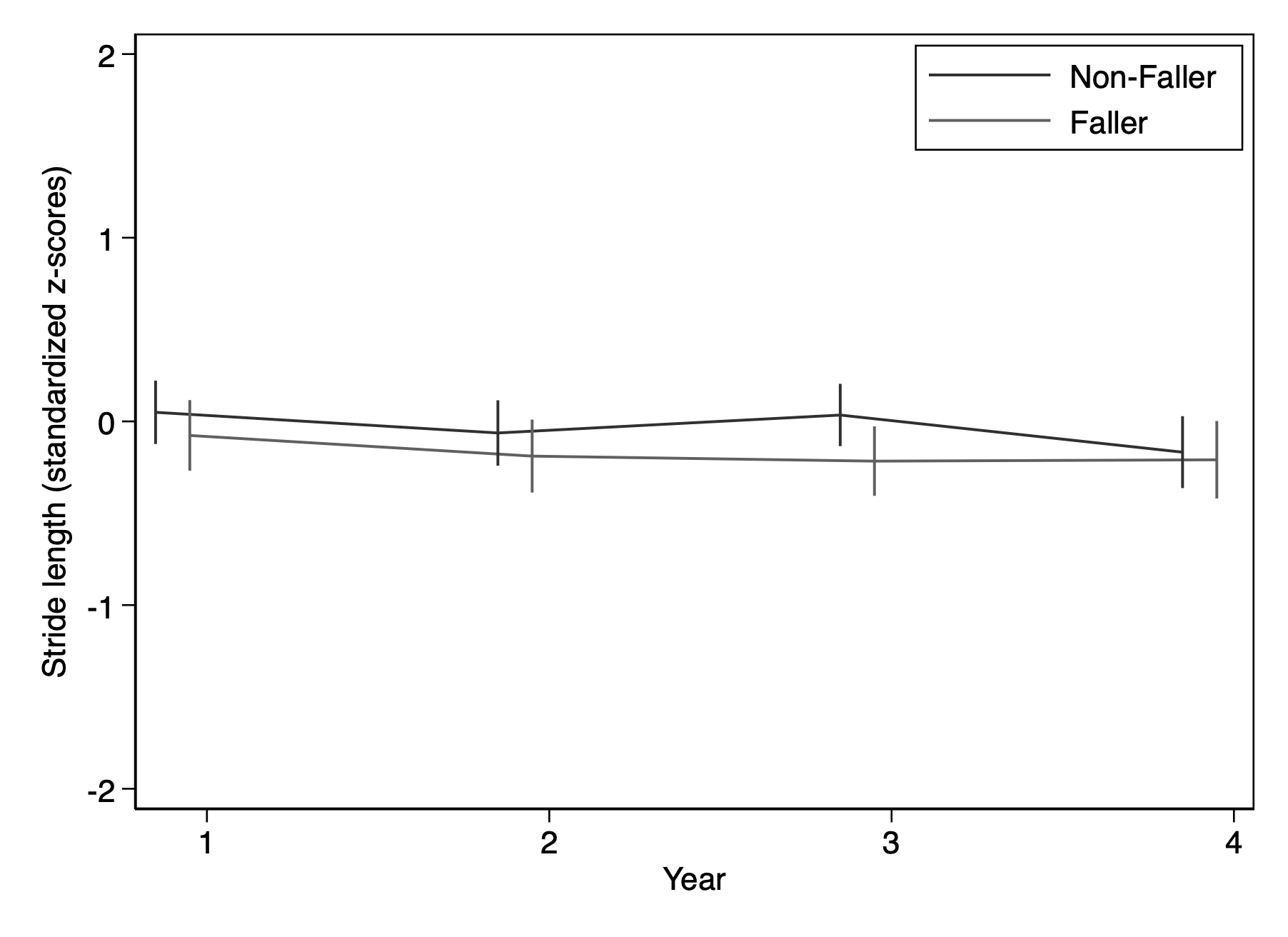

Supplement: Supplementary file 1 [file sensors-25-03712-s001.zip › FigureS2.jpg]

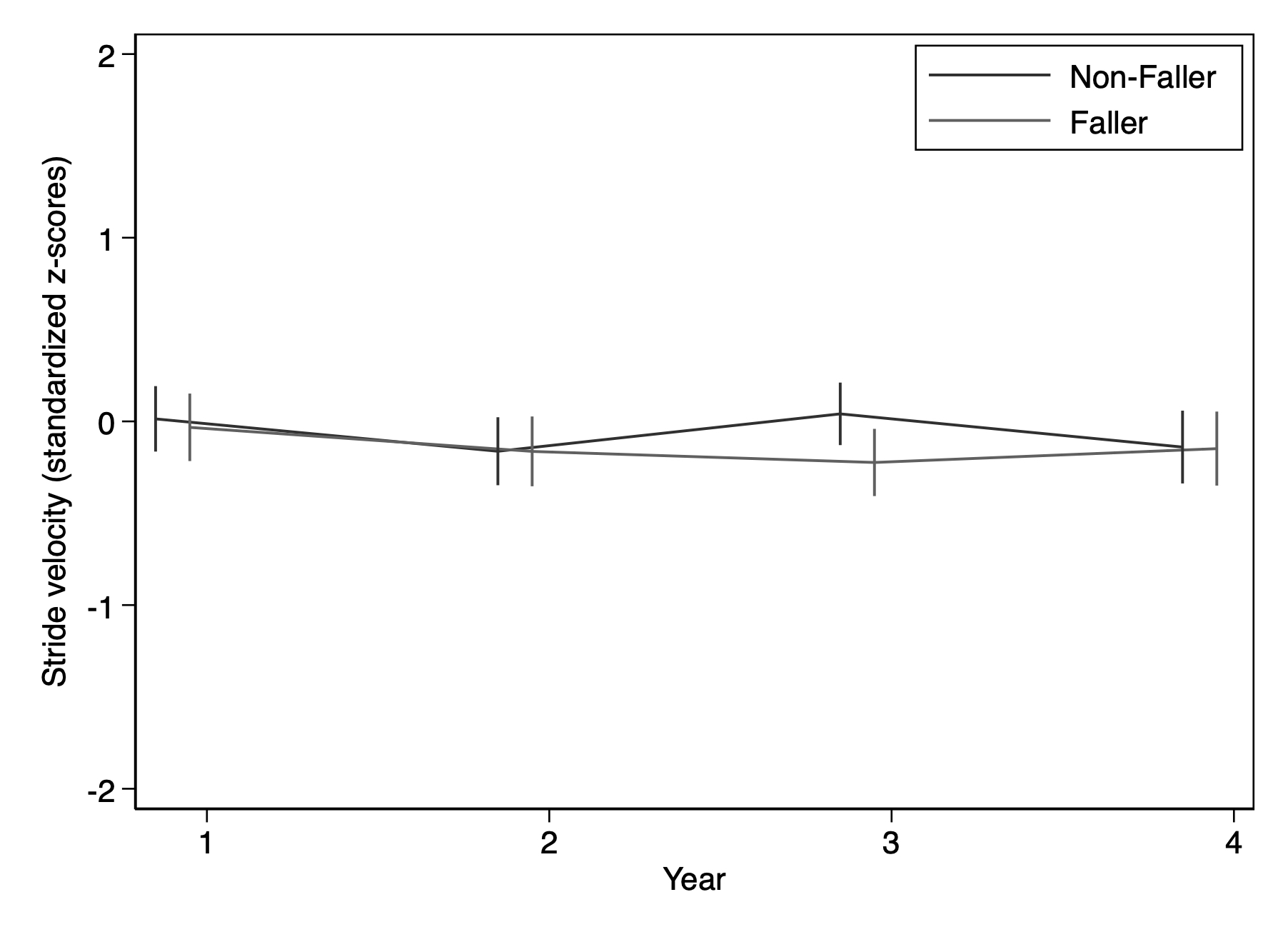

Supplement: Supplementary file 1 [file sensors-25-03712-s001.zip › FigureS3.jpg]

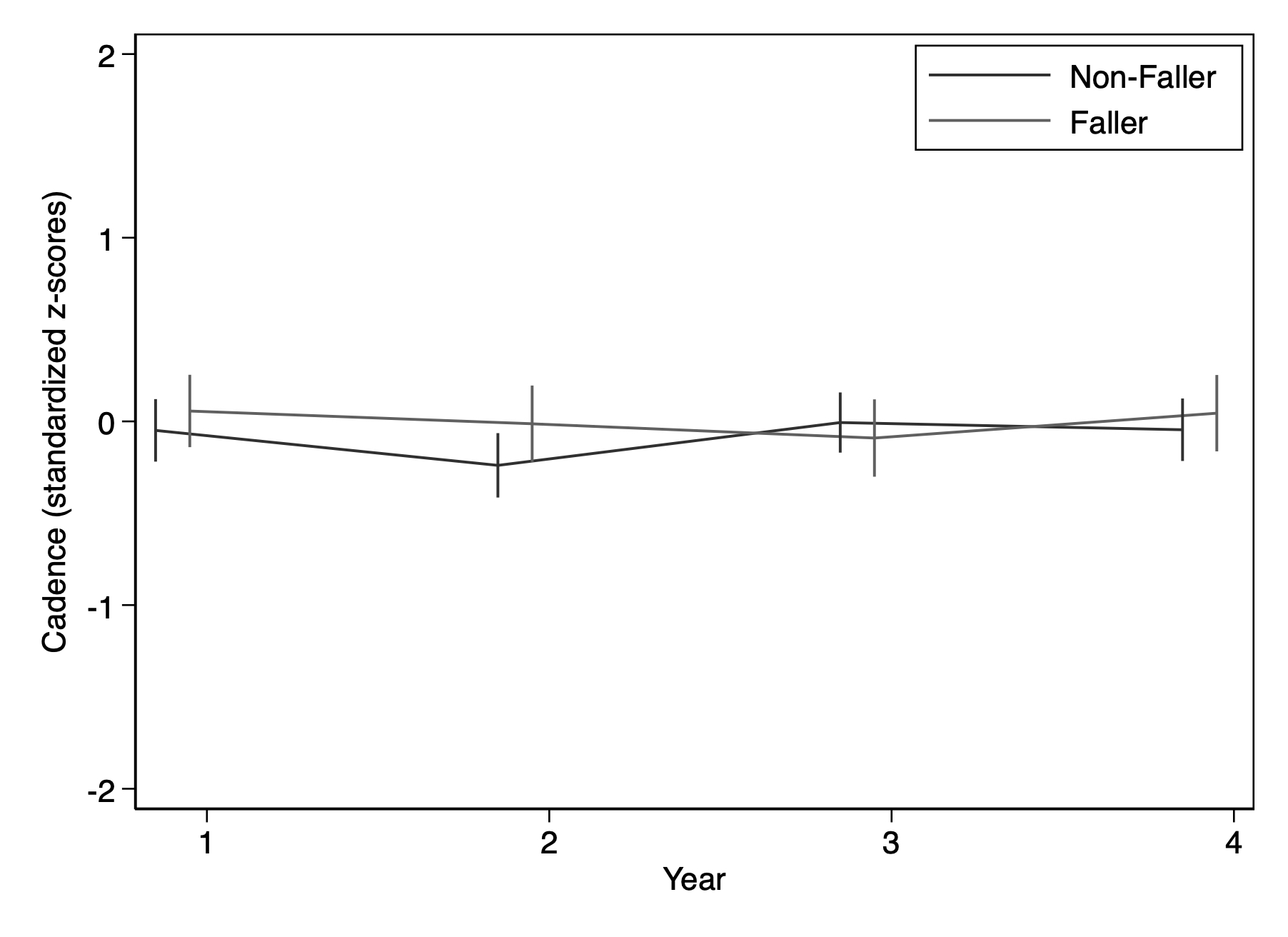

Supplement: Supplementary file 1 [file sensors-25-03712-s001.zip › FigureS4.jpg]
